# Retraction: The Irish National Alpha-1 Antitrypsin Deficiency (AATD) Registry: Examining the Prevalence of Radiological Abnormalities in a Population With Severe AATD

**DOI:** 10.7759/cureus.r231

**Published:** 2026-06-18

**Authors:** Amina Nameera

**Affiliations:** 1 School of Medicine, Royal College of Surgeons in Ireland, Dublin, IRL

The Editor-in-Chief has retracted this article due to the unauthorized publication of historical data from the National AATD Registry. The lead investigators of this study were unaware of its publication. The article contents have been suppressed to protect copyright.

